# Early Reconstructions of Complex Lower Extremity Battlefield Soft Tissue Wounds

**Published:** 2017-09

**Authors:** Ali Ebrahimi, Nasrin Nejadsarvari, Azin Ebrahimi, Hamid Reza Rasouli

**Affiliations:** 1 Department of Plastic Surgery, Baqiyatallah University of Medical Sciences, Tehran, Iran;; 2 Department of Medical Ethics, Iran University of Medical Sciences, Tehran, Iran;; 3 Tehran university of Medical Sciences, Tehran, Iran;; 4 Trauma Research Center, Baqiyatallah University of Medical Sciences, Tehran, Iran

**Keywords:** Wound, Lower extremity, Surgical flaps, Trauma, Battlefield, Soft tissue

## Abstract

**BACKGROUND:**

Severe lower extremity trauma as a devastating combat related injury is on the rise and this presents reconstructive surgeons with significant challenges to reach optimal cosmetic and functional outcomes. This study assessed early reconstructions of complex lower extremity battlefield soft tissue wounds.

**METHODS:**

This was a prospective case series study of battled field injured patients which was done in the Department of Plastic Surgery, Baqiyatallah University of Medical Sciences hospitals, Tehran, Iran between 2013-2015. In this survey, 73 patients were operated for reconstruction of lower extremity soft tissue defects due to battlefield injuries

**RESULTS:**

Seventy-three patients (65 men, 8 womens) ranging from 21-48 years old (mean: 35 years) were enrolled. Our study showed that early debridement and bone stabilization and later coverage of complex battlefields soft tissue wounds with suitable flaps and grafts of lower extremity were effective method for difficult wounds managements with less amputation and infections.

**CONCLUSION:**

Serial debridement and bone stabilization before early soft tissue reconstruction according to reconstructive ladder were shown to be essential steps.

## INTRODUCTION

Trauma places a massive burden on national economies and costs countries billions of dollars each year in healthcare system, law enforcement and productivity.^1^ Due to increased incidence of lower limb trauma, management of foot and ankle defects remains a common yet challenging reconstructive procedure. In this regard, optimal management of battlefield injuries to the lower extremity poses significant challenges to plastic surgeons. It is necessary to be a close cooperation between orthopedic and plastic surgeons for best management of battlefield injuries. Blast injuries are usually contaminated and composite in nature, concomitant trauma to vital organs usually exists and in time delivery to referral centers might also be significantly delayed due to the overwhelming number of the injured in the combat zone.^2^ Consequently, poor wound healing, chronic pain, heterotopic ossification and multiple drug resistant of wound infections are the leading complications associated with severe extremity injuries in the battlefield.^3^ Moreover, with the ever-growing advances of military technologies, more devastating and extensive war related injuries are on the rise.^4^

Satisfactory surgical outcomes are not just limited to cosmetic aspects and functional demands of the lower extremities must also be surgically addressed.^5^ Due to all these reconstructive difficulties and despite remarkable advancements of limb salvage procedures, in some cases amputation and use of advanced prosthetics remain a more feasible and beneficial alternative.^6^ Here, the challenges and proposed solutions regarding successful soft tissue reconstruction of the battlefield lower extremity trauma regarding to increase in terrorist attacks and increasing of civilian populations that are injured in such casualties were studied.

## MATERIAL AND METHODS

In a case series study of battlefield injured patients which was done in the Department of Plastic Surgery, Baqiyatallah University of Medical Sciences hospitals, Tehran, Iran between 2013-2015. All patients filled out the consent form before enrollment and the study protocol was approved by the Ethic Committee of Baqiyatallah University of Medical Sciences. In this survey, 73 patients were operated for reconstruction of lower extremity soft tissue defects due to battlefield injuries. 

**Fig. 1:**
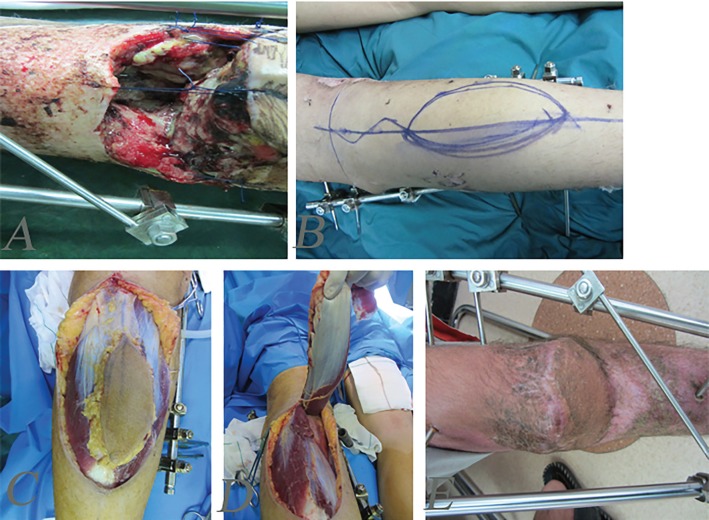
Complex defect of knee and patella and proximal tibia. **A:** Before reconstruction. **B:** Designing of medial gastrocnemius musculocutenous island flap. **C, D:** Intraoperative view of flap. E-delayed post-operation after three months

**Fig. 2:**
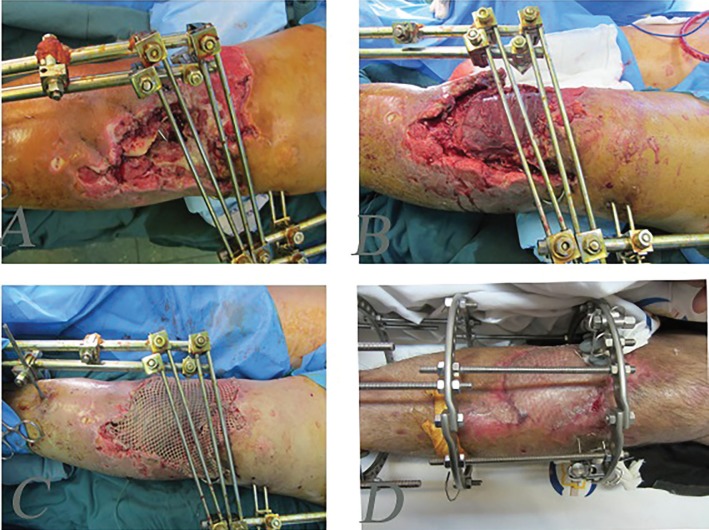
A 48 man after explosion injury and complex defect of proximal leg and bone defects. **A:** After stabilization of bone and debridement. **B:** Intraoperative view of lateral gastrocnemius musculocutenous flap. **C:** Early post operation with flap and skin graft. **D:** Delayed post operation after three months

**Fig. 3:**
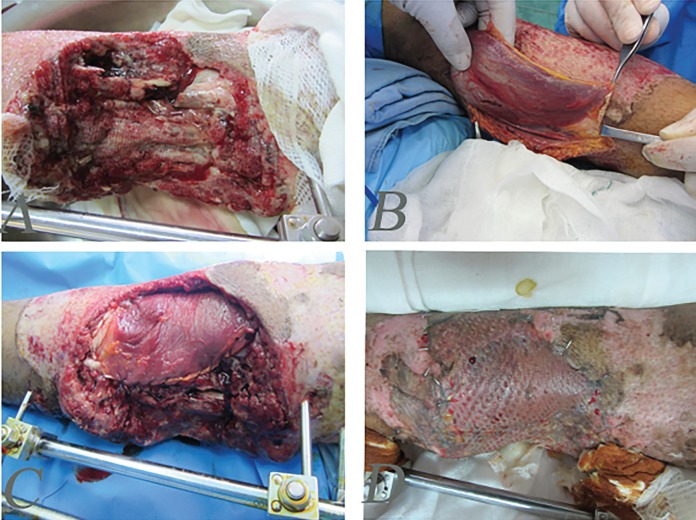
A 40 years old woman after explosion injury and complex defect of proximal leg soft tissue and bone. **A:** After stabilization of bone and debridement. **B, C:** Intraoperative view of medial gastrocnemius musculocutenous flap. **D:** One-month post-operation with reconstruction with flap and skin graft

The patients who referred for reconstruction of lower extremity defects were at the level of knee to foot. The characteristics of reconstructive procedures were shown in Table 1. According to anatomic site of injury, the options for complex wound reconstruction were different. Due to contamination in explosive wounds, it is necessary to do debridement several times before any reconstructive procedure, also the bone fractures must be stabilized prior to soft tissue coverage. The medial and lateral gastercnimus musculocutenous flaps was a good choice for knee (Figure 1) and proximal leg defects (Figures 2, 3). 

**Table 1:** Some characteristic of flaps and complex wounds location in lower extremity

**Type of flap**	**Location of defect**	**Number**	**Percent**	**Combined flap with skin graft**
Medial gastrecnimus	Knee and proximal leg	12	16.4	12
Lateral gastrecnimus	Knee and proximal leg	4	5.4	4
Revers sural	Wrist and foot	18	24.6	18
Soleus	Midleg	16	21.9	16
Superior maleolar	Distal leg	12	16.4	12
Toe fillet	Foot	11	15	6
Total		73	100	68

**Fig. 4:**
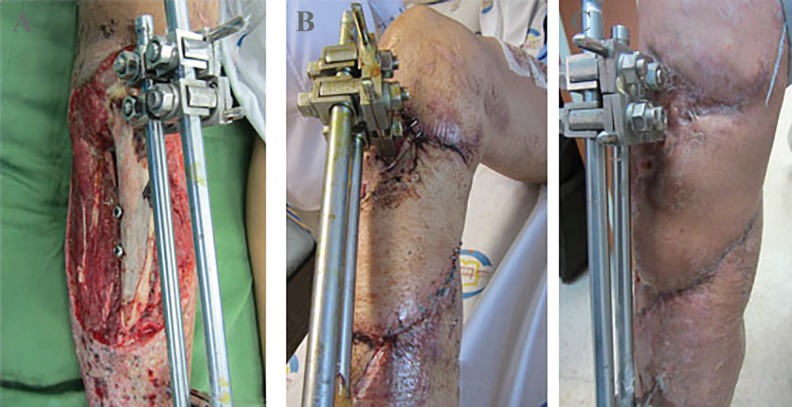
A 40 years old man with complex injury of bone and soft tissues of the leg. **A:** The patient after debridement and fixation with external fixator. **B:** The patient after one-month soft tissue reconstruction with combination of medial gastrecnimus fasiocutenous, soleus muscle flap and skin graft. **C:** Three-month post-operation

**Fig. 5:**
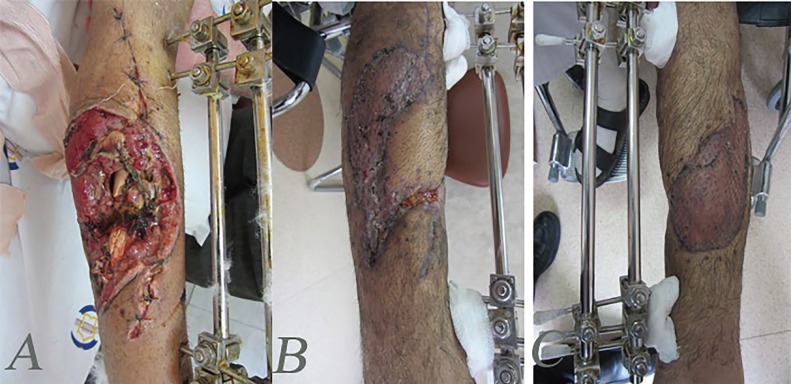
A 33 years old man with complex soft tissue and bone defects of midleg. **A:** Stabilization of bones before reconstruction with soleus flap. **B, C:** Three months after reconstruction with soleus muscle flap and skin graft

**Fig. 6:**
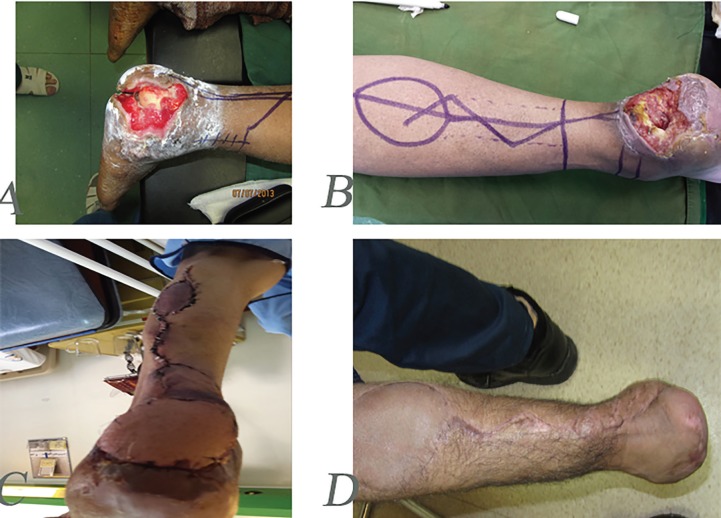
A 60 years old farmer with mine explosion injury and complex defect of heel. **A:** After debridement **B:** Designing of revers sural flap. **C:** One-month post operation and reconstruction with flap. **D:** One-year post-operation and natural gate

**Fig. 7:**
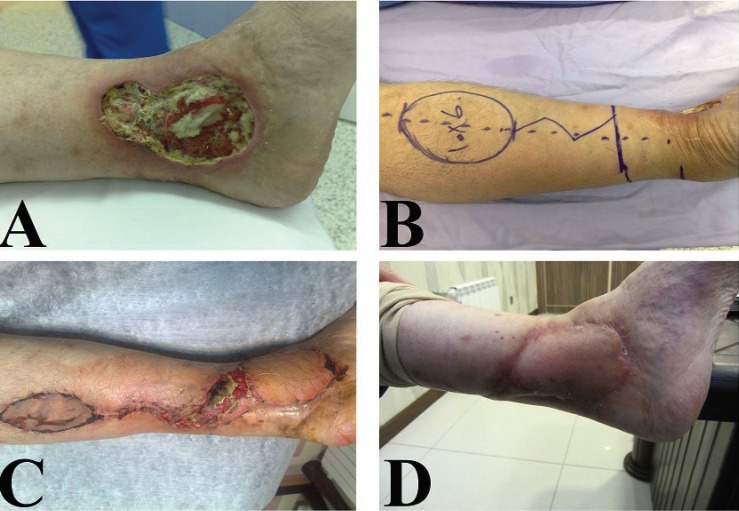
A 50 years old man with chronic explosion defect of medial distal leg. **A:** Before operation. **B:** Intraoperative view of revers sural flap designing. **C:** Early postoperative view. **D:** Delayed post-operation after six months

**Fig. 8:**
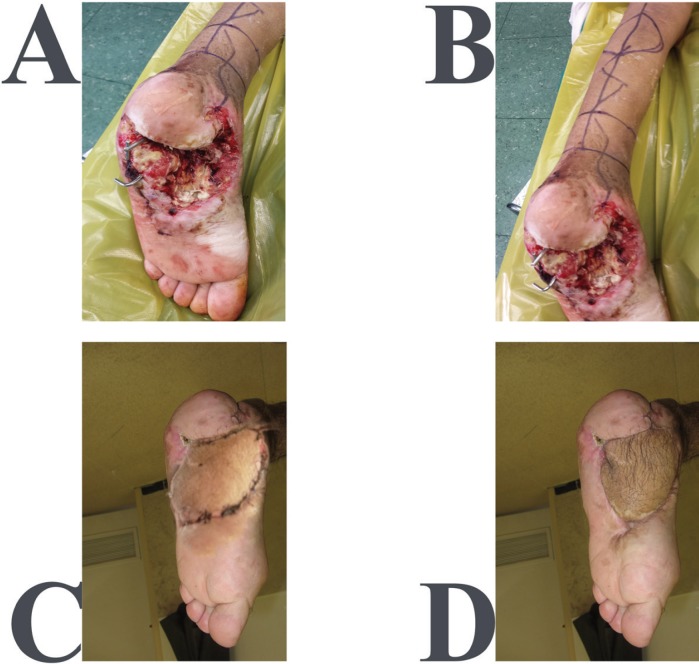
A 20 years old man with complex defect of hind foot and midfoot. **A: **Before operation **B:** Intraoperative designing of revers sural flap and bone stabilization view. **C:** Early post-operation. **D: **Delayed post-operation after six months

**Fig. 9:**
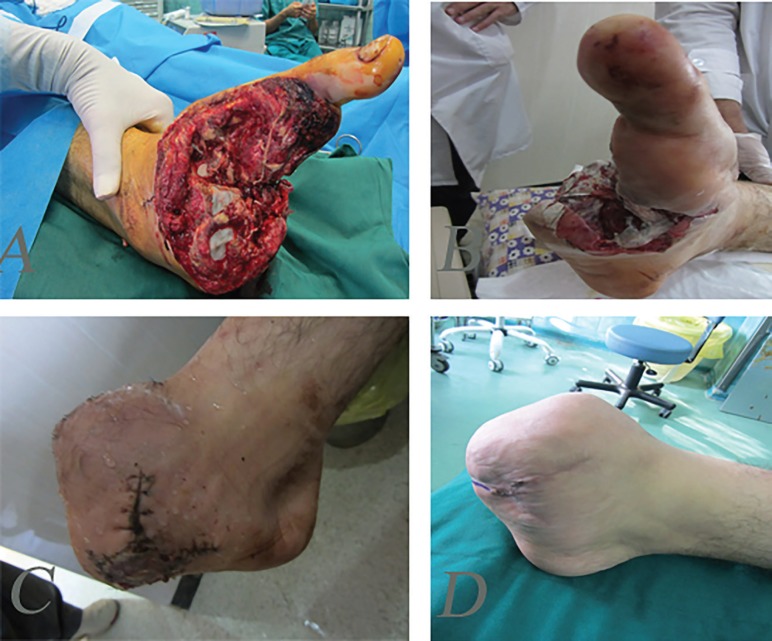
A 20 years old man after mine explosion injury and forefoot amputation. **A, B:** Before reconstruction. **C:** One-month after reconstruction of forefoot with toe fillet flap and skin graft. **D:** One-year post-operation

In some situations, blood supply of this muscle was damaged and in these conditions, fasiocutenous gastrecnimus flap was used in complex wounds and the result was good (Figure 4). In mid leg defects with bone exposure a combination of soleus muscle flap and skin graft had satisfactory results (Figure 5). In the lower third of leg and wrist complex defects, a variety of coverages were used, including revers sural flap (Figures 6, 7), superior maleolar flap, and free flaps. A good choice for hind foot and mid foot reconstructions was sural flap with functional results (Figure 8). For difficult partial forefoot defects, toe fillet flaps instead of foot amputation had better functional outcomes (Figure 9).

## RESULTS

Seventy-three patients (65 men, 8 women) of 21-48 years old (mean: 35 years) were included in this study. Our study showed that early debridement and later bone stabilization coverage of complex battlefields soft tissue wounds of the lower extremity were effective method for complicated wounds management with less amputation and infections. Also, in any anatomic defects special vascularized flaps near to defects with or without skin grafts revealed good results (Table 1). In addition, in some complicated cases that blood supply of gastrecnimus flap was damaged, fasiocutenous gasrecnimus flap (Figure 4) provided good blood supply and resulted in proximal leg defects with bone exposure. 

For reconstruction of wrist and proximal foot complex wounds, reverse sural flap was accompanied with functional results, and for complicated forefoot defects, toe fillet flap instead of foot amputation lead to better functional results. No significant complications (infection, osteomyelitis or amputation) after early managements of these complex lower extremity battlefield injuries during our survey were noticed. It is necessary to undertake a close cooperation between orthopedic and plastic surgeons for best management of battlefield injuries because these wounds were complex (contaminated and composite in nature) and were different from usual civilian accidents.

## DISCUSSION

Compared with the civilian injuries, combat related trauma has been reported with significant worse salvage outcomes.^7^ Currently, no reliable scoring system exists to predict the success rate of limb salvage procedures in management of foot and ankle war injuries. The reliability of scoring systems like Mangled Extremity Severity Score (MESS) to predict the likelihood of limb salvage has been questioned.^7^ In a large prospective study, the clinical utility of different lower-extremity injury-severity scoring systems was assessed in 556 patients. The predictive power of MESS, the Nerve Injury, Ischemia, Soft-Tissue Injury, Skeletal Injury, Shock, and Age of Patient Score (NISSSA), the Limb Salvage Index (LSI), the Hannover Fracture Scale-97 (HFS-97) and the Predictive Salvage Index (PSI) were compared. The researchers reported low sensitivity and suggested that these scoring systems should be used with caution when deciding the fate of limbs damaged by high-energy mechanisms.^8^

In contrast with these findings, a number of studies with smaller sample size or retrospective design indicated a higher sensitivity and specificity of MESS as a simple and available scoring system.^9^ In most cases and in absence of contraindications, it is logical to try salvation techniques initially. Decisions about amputation could be later finalized, considering the functional outcomes, patients’ preferences and cost-effectiveness analyses.^10^ Strategies to prevent wound infection and preservation of the remaining viable structures should be seriously taken into consideration in each reconstructive step. Early, extensive and serial wound debridement remains a mainstay of successful soft tissue reconstruction of war injuries. Due to the high-energy mechanism and high risk of contamination of blast injuries, tissues tend to devitalize and the reconstruction efforts have to be delayed enough to allow for sequential and aggressive debridement.^11^

We did early management of these wounds with good results. Despite possible problems in detecting perfusion and viability of the affected tissues, some experts suggested use of tourniquets in order to minimize excessive blood loss in serial debridements. However, tourniquet must be released at the end of each debridement procedure to reassess the remaining tissues and the need for further debridement.^12^ Debridement is very important in these injuries, our preferred method is serial conservative debridement until cleaning and good vascularized bed for reconstruction, this is especially important for early skin graft coverage. 

In wounds with bone exposure or more complicated wounds that need the vascularized flap for coverage, debridement is also important; but in these wounds, early coverage with flap is very important due to prevention of secondary complications such as osteomyelitis. Another important point is fixation and stabilization of any bone injury before soft tissue reconstruction, of course; secondary procedures on bone such as bone graft must be conducted after good soft tissue coverage and healing of the wounds (Figure 1). 

The timing and reconstruction success rate of combat related injuries of the lower extremity were previously determined showing that acceptable infection rates and low rates of flap loss, despite reconstruction of wounds in the sub-acute period within one week and three months of injury.^13^ we did not use a delay reconstruction in our cases. Important factors that determined the timing and type of reconstructive procedures in lower extremity war injuries were hemodynamic stability, concomitant trauma, exposed vascular structures and severity of the injury. Providing rapid cover for the repaired vascular structures using myocutaneous or muscle flaps was of great importance.^14^

There are several procedures for distal lower extremity reconstruction. In some cases, primary repair after debridement was the only surgery that was needed in superficial soft tissue injuries spit thickness skin grafts that were harvested preferably from intact limb as a good choice, but in some cases; combined procedures including skin grafts and some variety of flaps were necessary. Nerve damage usually can manage at the same time of soft tissue reconstruction or delayed repair and is dependent to wound condition and severity of damage.^15^

A variety of pedicled and free flaps could be used to cover the lower extremity war wounds. Reverse sural fasciocutaneous flap as a safe and quick pedicled flap requires no microvascular expertise. This flap is based on sural vessels and is a versatile option covering the lower limb wounds particularly in patients suffering multiple trauma or vascular pathologies.^15^ A recent systematic review of the literature reported trauma as the most common indication of the distally based sural fasciocutaneous flap. This flap has been used most frequently to cover heel, ankle and foot defects, respectively.^16^

Rectus abdominis myocutaneous flaps have historically remained a mainstay of reconstructive procedures in lower abdomen, groin and anterior thigh defects. However, alternatives including pedicled anterolateral thigh flap have been reported as superior alternatives with promising results and less donor site morbidity.^17^ Gastrocnemius and soleus flaps could be used to cover the wounds around the knee and in cases of extensive damage to the lower extremity, a combination of both flaps have been recommended.^18^ We have shown combination of medial gastrocnemius faciocutenous and soleus muscle flap as vascularized flaps for large proximal leg defect with bone exposure due to explosion injured patient, reversed superficial peroneal neurocutaneous island flap that has been reported as an excellent alternative flap in soft tissue reconstruction of the ankle and foot as previously described.^19^

We explain soft tissue reconstruction of the distal lower limb defects in four parts. These parts are forefoot, midfoot, hindfoot and ankle-distal leg. The reconstructive surgeon have to consider the reconstructive ladder for any surgery in these parts, this means that simple procedures have complex reconstructions according to severity of blast trauma (primary repair, local flaps, skin grafts, regional and free flaps). Combat related trauma of the forefoot usually leads to complete or partial amputation of toes. Various reconstructive techniques according to reconstructive ladder could be used based on the toes involved to reach excellent functional and cosmetic outcomes. Big toe reconstruction is of value in maintaining good stability during gate and should be considered a priority. Free peroneal and scapular flaps have been tried with acceptable cosmetic and functional outcomes in post-traumatic reconstruction of the big toe.^20^

Also, use of free vascularized bone graft of the supracondylar region of the femur has been described with excellent outcome in cases of segmental bone defects of the great toe.^21^ Dorsalis Pedis Adipofascial Perforator (DPAP) flap has been anatomically studied as another possible alternative in big toe reconstruction. Yet, its effectiveness remains to be verified by further clinical studies.^22^ Amputation is frequently preferred over the salvage of the lesser toe to prevent unnecessary extra challenge to the surgical team. However, reconstructive techniques using autogenous bone grafting with external fixation exist with satisfactory outcomes were reported in case series.^23^

Use of autologous hemi-toe osteochondral graft is another possibility with excellent reported functional improvements in reconstruction of interphalangeal joints.^24^ Another important issue in reconstruction of the forefoot defects is maintaining the dorsal foot and ankle contour for shoe fitting. In order to address this issue, distally based adipofascial flap with skin graft could be utilized with minimum donor site morbidity, optimal aesthetic and functional results.^25^ Distally based lateral supramalleolar adipofascial flap has also been used to cover the dorsal foot and ankle surface in this regard.^26^

Midfoot blast injuries might result in open comminuted fractures and high potential of serious complications should be considered before selecting the ideal surgical approach. Open reduction and internal fixation is the standard approach in management of severe tarsometatarsal fractures of civilian foot and ankle trauma.^27^ Yet, as previously discussed, a combination of factors complicate management of combat related trauma and as a result, optimal techniques used in the civilian setting might not properly work in soft tissue reconstruction of war injuries.^10^

External fixation as an alternative with satisfactory short and long-term outcomes is commonly practiced in military medicine in reconstruction of the severe open ankle and foot fractures in combat zone. This technique is superior to the conventional civilian approach regarding its applicability in face of poor recourses and less surgical insult to the severely damaged soft tissues.^28^ However, some reports suggest persistent morbidity despite proper and in time use of external fixation in midfoot crush injuries.^29^ In another approach, successful use of saphenous cross-leg flap with modified Masquelet technique in reconstruction of extensive medioplantar defect was reported. Following saphenous cross-leg flap, corticocancellous iliac bone graft was used to reconstruct the medial cuneiform deformities.^30^

In addition, a viable soft tissue coverage of the distal lower limb was reverse sural artery fasciocutaneous flap that has been reported with acceptable outcomes in carefully selected patients.^31^ The hind foot defects are the most difficult to reconstruct successfully and are infamous with high rates of complications and final amputation, particularly if open calcaneal fracture is present. Informed early decision about amputation should be discussed with the patients before reconstruction procedures; as many limb salvage efforts would fail in these patients due to poor functional outcomes, osteomyelitis and infection. Size, location, fracture type and severity in addition to mechanism of injury could predict amputation risk in war-related calcaneal fractures. Amputation could lead to improved activity levels and reduced pain compared with the demanding salvage protocols in these cases.^32^

A special pattern of hind foot trauma faced in war zone is caused by anti-vehicle mines and is referred to as “deck-slap” injury. Review of the literature highlights devastating nature of these injuries and low chance of returning to military duties due to poor functional outcomes.^33^ Finally, if patient prefers limb salvage gracilis or suralis flaps with free skin grafts could be considered for soft tissue reconstruction of the calcaneal fractures.^34^ we have also shown reconstruction of complex hindfoot wound after mine explosion defect with reverse sural flap (Figure 5).

 In reconstruction of ankle and distal leg, posterior tibial perforator flap, revers sural flap and lateral supramalleolar skin flap based on supramalleolar artery provided a wide variety of reconstructive options in management of the distal lower limb soft-tissue defects. Use of free flaps is more reliable than distally based fasciocutaneous flaps in patients with poor vascularity of the lower limb or in cases with extensive defects.^35^ We used medial gastrecnimus free flap in complex heel wound due to mine explosion. 

As discussed earlier, reverse sural flap is considered a versatile option in optimal management of lower leg defects especially in maleolar defects, heel reconstruction, hindfoot and also midfoot reconstructions, we used this flap frequently for these reconstructions. This flap has a large arc of rotation and could be safely used, free of significant pressure to cover defects that are more extensive.^36^ Soft tissue reconstruction of combat related lower extremity injuries poses significant challenges to the plastic and orthopedic surgeons. In addition, great patience and expertise are necessary to achieve most satisfactory aesthetic and functional patient-oriented outcomes. So serial debridement and bone stabilization before early soft tissue reconstruction according to reconstructive ladder is an essential step. 
